# Survival Analysis Based on Clinicopathological Data from a Single Institution: Chemotherapy Intensity Would Be Enhanced in Patients with Positive Hormone Receptors and Positive HER2 in China Who Cannot Afford the Target Therapy

**DOI:** 10.1155/2013/606398

**Published:** 2013-07-30

**Authors:** Jianyi Li, Shi Jia, Wenhai Zhang, Yang Zhang, Xiang Fei, Rui Tian

**Affiliations:** Department of Breast Surgery, Shengjing Hospital of China Medical University, Sanhao Street No. 36, Heping District, Shenyang, Liaoning 110004, China

## Abstract

*Background*. Immunohistochemical markers were often used to classify breast cancer into subtypes. The aim of this study was to estimate death and tumor progression for patients with the major subtypes of breast cancer as classified using immunohistochemical assay and to investigate the patterns of benefit from the therapies over the past years. *Methods*. The study population included primary, operable 199 invasive ductal breast cancer patients, with the median age of 51.1 years old. All patients underwent local and/or systemic treatments. The clinicopathological characteristics and clinical outcomes were retrospectively reviewed. The expression of estrogen receptor, progesterone receptor, human epidermal growth factor receptor 2, and Ki67 was analyzed by immunohistochemistry. All patients were classified into the following categories: luminal A, luminal B, HER2 overexpression, and triple-negative subtypes. *Result*. The median follow-up time was 33 months. Luminal A tumors had the lowest rate of tumor progression (0%, *P* = 0.006), while luminal B, HER2 over-expression, and triple-negative subtypes were associated with an increased risk of tumor progression (15.4, 19.2, 15.4%). Clinicopathological subtypes retained independent prognostic significance (*P* = 0.008). There were significant differences by Cox model analyzed in age, menopause, lymph node metastasis, and HER2 for the event of death and tumor progression (*P* < 0.05), and there were significant differences only in chemotherapy for the event, respectively (*P* < 0.05). *Conclusion*. Clinicopathological subtypes of breast cancer could robustly identify the risk of death and tumor progression and were significant in making therapeutic decision. HER2 was the important poor indicator. The chemotherapy intensity would be enhanced for patients with luminal B, especially for HER2 over-expression subgroup.

## 1. Introduction

The 12th St. Gallen International Breast Cancer Conference (2011) Expert Panel adopted a new approach towards classifying patients for therapeutic purposes based on the recognition of intrinsic biological subtypes within the breast cancer spectrum. For practical purposes, these subtypes may be approximated using clinicopathological factors rather than gene expression array criteria, including luminal A (LA) and luminal B (LB) breast tumors, overexpression of human epidermal growth factor receptor 2 (HER2), and triple-negative breast cancer (TNBC) [1]. Clinicopathological subtypes are becoming increasingly relevant to both the diagnosis of breast cancer patients with different prognoses and their therapies [2]. Hormone receptor-positive breast cancer is a factor that is predictive of the response to endocrine therapy, and HER2-positive breast cancer is sensitive to targeted therapy with a specific monoclonal antibody [3]. In fact, endocrine therapy has fared better than targeted therapy in developing countries such as China because of economic reasons. A recent study suggested that one year of adjuvant trastuzumab treatment was cost-effective, and the incremental cost for an additional year of life was more than US$7,500 in developed areas such as Beijing, Shanghai, and Guangzhou [4]. However, the economic burden of US$40,000, which is the standard cost of chemotherapy, was very expensive for most patients in underdeveloped areas. The Department of Breast Surgery was established in April 2005, and the follow-up system was established in June 2006. In addition, the number of patients treated per year increased from 40 cases in 2005 to 440 cases in 2011. Unfortunately, the first patients with HER2 overexpression were not accepted to receive targeted therapy until May 2010. The data of same period patients with no target therapy was provided naturally. The aim of this study was to estimate the death and relapse rates of patients with major subtypes of breast invasive ductal cancer as classified using immunohistochemical assays; In addition, we investigated beneficial treatment patterns from therapies given over the past several years. 

## 2. Materials and Methods

### 2.1. Study Population

Between June 2006 and December 2009, a total of 199 patients with invasive breast cancer were evaluated in the Department of Breast Surgery at the Shengjing Hospital of China Medical University, Shenyang, China. All patients were treated with adjuvant systemic therapy (chemotherapy, radiotherapy, and endocrine therapy) guided by the National Comprehensive Cancer Network (NCCN). However, all the patients with positive HER2 did not accept the target therapy. The followup was carried out at 3-month intervals during the first two years, at 6-month intervals during the next three years, and at 12-month intervals thereafter. The initial followup began in December 2011. The diagnosis of local recurrence and contralateral breast cancer was supported by biopsy, and distant metastasis was diagnosed by more than two kinds of imaging examinations. The outcome criteria were relapse and death due to disease, time until progression (disease-free survival, DFS), and overall survival (OS). In this rather homogeneous and relatively small group of patients, we collected anthropometric data (age at diagnosis, menopause, family history, and hormonal therapy), as well as variables related to the tumor (size, location, TNM staging, histologic grade, and lymphovascular invasion). Pathological tumor stage was assessed according to the criteria established by the 6th edition of the American Joint Committee on Cancer (AJCC) staging manual. The histological grade of the tumors was classified into grades I–III according to the Nottingham combined histological grade.

### 2.2. Immunohistochemistry and Fluorescence In Situ Hybridization

Immunohistochemistry (IHC) was performed on formalin-fixed, paraffin-embedded samples obtained from the pathology registry. Tissue sections (5 *μ*m) were deparaffinized in xylene and rehydrated in a graded series of ethanol. Slides were treated with methanol containing 0.3% hydrogen peroxide to block any endogenous peroxidase activity. Heat-mediated antigen retrieval with the pressure cooker method was used for all stainings except for the epidermal growth factor receptor (EGFR), which did not need retrieval. Antibodies recognizing the estrogen receptor (ER), progesterone receptor PgR, and HER2 were used for immunohistochemical studies on serial tissue sections from each case; EGFR and Ki67 antibodies were used in luminal A tumors. Thus, in total, five markers were assessed, namely, ER, PgR, HER2, and EGFR, which were used for breast carcinoma subtypes, and Ki67, which was used to divide luminal A tumors into two groups. The primary antibodies used in this study included ER (SP1, 1 : 200 dilution; ZETA), PgR (SP2, 1 : 200 dilution; ZETA), HER2 (CB11, 1 : 100 dilution; Invitrogen, Carlsbad, CA, USA), EGFR (SP9, 1 : 100 dilution; Invitrogen), and Ki67 (K-2, 1 : 100 dilution; Invitrogen). Immunostaining was scored in a double-blinded manner by two different pathologists who were blinded to the clinicopathologic characteristics and outcome of each patients. For each antibody, the location of immunoreactivity, percentage of stained cells, and intensity were determined. The evaluation of protein expression was determined from the mean of each individual case. ER and PgR staining was assessed by Allred scoring, with positive scores ranging from 2 to 8 [5]. EGFR staining was considered positive if any cytoplasmic and/or membrane staining was observed, HER+ (IHC) was defined as a strong whole membrane staining in >30% of the tumor cells, and Ki67 status was expressed in terms of percentage of positive cells, with a threshold of 14% of positive cells [6]. Fluorescence in situ hybridization (FISH) analysis was performed on IHC+ tumors using the PathVysion HER2 DNA Probe Kit (Vysis, Downers Grove, IL, USA). HER2-positive staining was defined as FISH positive, and HER2-negative staining was defined as IHC 0 or negative FISH results.

### 2.3. Clinicopathological Subtypes

The clinicopathological subtypes of breast cancer were previously described and were best matched with gene expression patterns [1]. Briefly, the subtype definitions are as follows: luminal A (ER+ and/or PgR+ and HER2– and/or Ki67 < 14%), luminal B (ER+ and/or PgR+ and HER2+ and/or Ki67 ≥ 14%), HER2 overexpression (ER–, PgR–, and HER2+), and triple negative (ER–, PgR–, HER2–) (Table 1).

### 2.4. Statistics

All statistical analyses were carried out using SPSS software (version 17.0 for Windows). The correlation analyses between clinicopathological subtypes and the various biological factors were examined by the *X* ^2^ test or ANVOA analysis. For survival analysis, overall survival (OS) and disease-free survival (DFS) were conducted using the Kaplan-Meier curves. The log-rank test was used to compare survival differences among the subtypes. Cox proportional hazards models were used to calculate relative risk accounting for covariates. *P* values less than 0.05 were considered statistically significant.

## 3. Results

### 3.1. Clinicopathological Subtypes and Profiles of Patients

Sixty-six (33.2%) patients were classified as luminal A, 55 (27.6%) were luminal B, 26 (13.1%) were HER2-positive, and 52 (26.1%) had triple-negative breast cancer. The distribution of characteristics among the various intrinsic subtypes of breast cancer is listed in Table 2; there were significant differences in histological grade, chemotherapy time, chemotherapy program, chemotherapy cycle, radiotherapy, and endocrine therapy among the subtype cohorts (*P* < 0.05). Of the 199 patients involved in this study, 181 (91%) were treated with mastectomy, 18 (9%) were treated with tumorectomy, 94.5% received chemotherapy, 43.7% received radiotherapy, and 57.8% received hormone therapy (Table 2).

### 3.2. Followup and Survival Analysis

**Figure 1:**
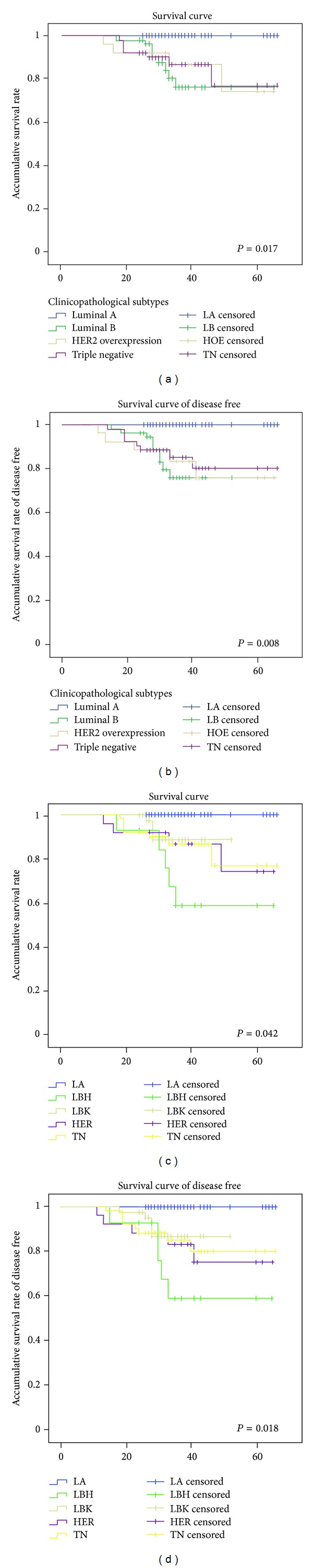
(a) show overall survival curve grouped by ER, PR, and Her 2; (b) show disease-free survival curve grouped by ER, PR, and HER2; (c) show overall survival curve grouped by ER, PR, HER 2, and Ki67; (d) show disease-free survival curve grouped by ER, PR, Ki67, and HER2.

With a follow-up period from 13 to 66 months, the actuarial OS of luminal A, luminal B, HER2 overexpression, and triple-negative breast cancer was 100, 85.5, 84.6, and 86.5%, respectively, and there were significant differences among the subtypes (*P* = 0.015) (Table 3). The median survival time of patients with luminal A, luminal B, HER2 overexpression, and triple-negative subtypes of breast cancer was 66, 60, 60, and 66 months, respectively, and there were significant differences among subtypes (*P* = 0.033) (Table 3). In particular, significant differences were on the overall survival curve (*P* = 0.017) (Figure 1(a)). The actuarial DFS of luminal A, luminal B, HER2 overexpression, and triple-negative subtypes was 100, 83.6, 80.8, and 84.6%, respectively, and there were significant differences among the subtypes (*P* = 0.006) (Table 3). The median disease-free survival time of luminal A, luminal B, HER2 overexpression, and triple-negative subtypes was 66, 60, 60, and 66 months, and there were significant differences among the subtypes (*P* = 0.013) (Table 3), particularly on the disease-free survival curve (*P* = 0.008) (Figure 1(b)). 

### 3.3. Cox Proportional Hazards Model

Twelve kinds of patient's independent biological factors were used to build a Cox proportional hazard model for death and tumor progression (Table 4). There were significant differences in age, menopause status, lymph node metastasis, and HER2 overexpression for death (*P* < 0.05) and Exp (*B*) (expose of the *B* coefficient), namely, 1.01, 5.28, 10.01, and 4.32, respectively. There were significant differences in age, menopause status, lymph node metastasis, and HER2 overexpression for tumor progression (*P* < 0.05) and Exp (*B*), namely, 1.08, 4.45, 6.97, and 3.88, respectively. Four kinds of independent treatment factors were used to build a COX proportional hazard model for death and tumor progression. There were significant differences only in chemotherapy (*P* < 0.05) and Exp (*B*), which were 1.63 for death and 1.60 for tumor progression (Table 4).

## 4. Discussion

Breast cancer remains one of the leading causes of cancer-related death worldwide, with invasive ductal cancer (IDC) being the most common type [7]. However, the prognosis significantly changed with the introduction of adjuvant therapy, including chemotherapy, radiotherapy, endocrine therapy, and targeted therapy [8]. Breast cancer is a heterogeneous group of neoplasms, and the aim of grouping them into clinicopathological subtypes was to predict prognosis and guide treatment [1]. Recent studies have shown that patients with luminal A IDC have the best prognosis among all subtypes, are sensitive to endocrine therapy, and are insensitive to chemotherapy [9]. In that study, none of the 66 patients with luminal A IDC died or experienced tumor progression, the OS and DFS were 100%, and the median survival and disease-free survival time were 66 months. Most experts who participated in the St. Gallen consensus considered the endocrine therapy enough for patients with early luminal A breast cancer [1]. Triple-negative tumors have two subgroups: basal-like and normal, with the expression of cytokeratin being the main difference between them [10]. Previous studies demonstrated that triple-negative breast cancer is associated with young age, high-grade tumors, advanced stage at diagnosis, difference in chemotherapy response compared to other subtypes, and the shortest survival [11]. Our data suggest that patients with triple-negative breast cancer do not have the worst prognosis, which related with confounding factors of normal like subgroup. Of course, the factors that affected the results of the survival analysis were complex, and the main reason could be confounding factors of normal-like subtypes. Survival analysis showed that patients with HER2 overexpression had the worst OS and DFS, with patients with luminal B tumors ranking second. In fact, it appears that a lack of anti-HER2 therapy (targets therapy of trastuzumab) was the main reason that patients had a poor prognosis. However, it was unclear why the prognosis of patients with luminal B tumors was worse than those with triple-negative breast cancer. According to the principle of clinicopathological subtypes, luminal B tumors can be divided into two subgroups: Ki67 ≥ 14% and HER2 overexpression [12]. We conducted survival analysis based on five subtypes; namely, luminal A, luminal BK (Ki67 ≥ 14%), luminal BH (HER2 overexpression), HER2 overexpression, and triple-negative breast cancer (Figures 1(c) and 1(d)). As Figures 1(c) and 1(d) show, the OS of patients with luminal BH (HER2 overexpression) was the lowest of all subtypes, and the risk of death was the highest of all subtypes (*P* = 0.042). DFS was the lowest of all subtypes, and risk of tumor progression was the highest of all subtypes (*P* = 0.018). This result was unexpected, which the prognosis of patients with luminal BH was poorer than those with HER2 subtype. It was not enough to interpret the differences between luminal BH and HER2 subtypes that no patients with HER2 enriched were accepted to targets therapy. Two kinds of Cox models were established to find the relative risk factor of death and tumor progression, including biological factors and treatment, respectively. Table 4 shows that age, menopause status, lymph node metastasis, and HER2 overexpression are significant relative risk factors for death and tumor progression. However, there was no significance among the four subtypes in age, menopause status, and lymph node metastasis among the subtypes (Table 2). Table 4 showed that chemotherapy was the only significant relative risk factor for death and tumor progression. In fact, there were significant differences among the four subtypes in adjuvant or neoadjuvant therapy, as well as the program and cycle of chemotherapy (Table 2). Therefore, we speculated that chemotherapy factors were the main reasons underlying the poorer prognosis of patients with luminal B tumors compared to other subtypes. Since the introduction of CMF (Cyclophosphamide + Methotrexate + 5-Fluorouracil) chemotherapy in the last century, chemotherapy has been at the core of adjuvant treatment [13]. Compared with CMF, 5-year Relapse Free Survival (RFS) and OS were favored anthracycline-containing regimens [14]. Based on the Breast Cancer International Research Group (BCIRG) 001 trial, the submitted evidence shows that TAC (Docetaxel + Doxorubicin + Cyclophosphamide) is associated with superior disease-free and overall survival at 5 years compared to the anthracycline-based regimen FAC (Doxorubicin + Cyclophosphamide + 5-fluorouracil), and the side effects from TAC were more serious than those from FAC [15]. In our study, the proportion of patients who accepted TAC chemotherapy was the least among patients with luminal B tumors compared to that of patients with other breast cancer subtypes (5.5%) (Table 2). Upon the review of the selection process about chemotherapy, it was in the main subjective reasons causing lower intensity of chemotherapy that the primary doctors consider that patients with luminal B subtypes were sensitive to endocrine therapy and had the good prognosis like the Luminal A subtype. The secondary reason was that the improved prognosis of patients with TNBC related with the chemotherapy protocols included docetaxel (61.5%) (Table 2).

In summary, our data confirmed that HER2 overexpression is an important indicator of poor prognosis. As a result, anti-HER2 therapy has clinical significance. Furthermore, our data prompted that chemotherapy intensity would be enhanced in patients with positive hormone receptors and positive HER2. These patients did not accept the target therapy because of economic reasons and usually were not accepted in intensive chemotherapy due to doctor's subjective deviations in China. Further investigation should be conducted in which chemotherapy program is more effective for them.

## Figures and Tables

**Table 1:** Clinicopathological subtypes definitions.

Parameters	Clinical-pathologic definition
Luminal A	ER and/or PgR positive and HER2 negative and Ki-67 low (<14%)
Luminal B	
HER2 negative	ER and/or PgR positive and HER2 negative and Ki-67 high (≥14%)
HER2 positive	ER and/or PgR positive and Any Ki-67 and HER2 overexpressed or amplified
HER2 overexpression	HER2 overexpressed or amplified and ER and PgR absent
Triple negative	ER and PgR absent and HER2 negative

**Table 2:** Parameters of patients (grouping by the clinicopathological subtypes).

Parameters	Luminal A (*n* = 66)	Luminal B (*n* = 55)	HER2+ (*n* = 26)	TNBC (*n* = 52)	Statistics (*F* or *X* ^2^)	*P*
Age (years)					0.838	0.475
Mean	51.38 ± 11.05	51.96 ± 9.41	48.27 ± 9.91	51.37 ± 9.76		
Range	29*∼*82	35*∼*75	2*∼*71	25*∼*70		
Menopause					2.119	0.548
Yes	26	24	7	21		
No	40	31	19	31		
Family history					1.063	0.786
No	53	47	20	42		
Cancer	9	6	4	9		
Breast cancer	4	2	2	1		
Quadrant					7.663	0.054
Areolar	5	1	4	1		
Outer upper	40	31	18	34		
Outer lower	8	12	1	5		
Inner lower	4	4	2	4		
Inner upper	9	7	1	8		
Operation					4.088	0.252
Mastectomy	62	50	21	48		
Tumorectomy	4	5	5	4		
Diameter	2.22 ± 1.40	2.30 ± 0.96	2.55 ± 1.42	2.70 ± 1.34	1.566	0.199
Histological grade					17.209	0.001
I	17	4	4	9		
II	46	43	15	22		
III	3	8	7	21		
Node metastasis					3.664	0.300
Yes	24	29	10	24		
No	42	26	16	28		
Cancer thrombosis					4.291	0.232
Yes	16	21	11	15		
No	50	34	15	37		
P53					6.865	0.076
Negative	33	18	15	28		
Positive	33	37	11	24		
Clinical stage					4.755	0.191
I	25	13	11	16		
IIA	25	20	5	16		
IIB	9	8	2	7		
IIIA	4	11	4	7		
IIIB	0	0	0	0		
IIIC	3	3	4	6		
IV	0	0	0	0		
Chemotherapy					11.395	0.010
No	9	1	1	0		
Adjuvant	54	52	20	48		
Neoadjuvant	3	2	5	4		
Program					15.533	0.001
No	9 (13.6%)	1 (1.8%)	1 (3.8%)	0 (0%)		
CMF	3 (4.5%)	2 (3.6%)	0 (0%)	2 (3.8%)		
CAF or AC	9 (13.6%)	5 (9.1%)	0 (0%)	7 (13.5%)		
CEF or EC	25 (37.9%)	37 (67.3%)	14 (53.8%)	11 (21.2%)		
TC or TP	8 (12.1%)	7 (12.7%)	9 (34.6%)	18 (34.6%)		
TAC or A-T	12 (18.2%)	3 (5.5%)	2 (7.7%)	14 (26.9%)		
Cycle	5.15 ± 2.25	5.78 ± 1.07	5.77 ± 1.18	5.92 ± 1.19	2.784	0.042
Radiotherapy					9.552	0.023
No	47	27	11	27		
Yes	19	28	15	25		
Endocrine therapy					155.813	0.000
No	5	1	26	52		
TAM	50	42	0	0		
AI	11	12	0	0		

Illustration: the ages, diameter of tumor, and chemotherapy cycle were metric data and examined by ANVOA analysis; other digital parameters were examined by *X* ^2^ test.

**Table 3:** Survival analysis.

Parameters	Luminal A (*n* = 66)	Luminal B (*n* = 55)	HER2+ (*n* = 26)	TNBC (*n* = 52)	Statistics (*F* or *X* ^2^)	*P*
Overall survival	66 (100%)	47 (85.5%)	22 (84.6%)	45 (86.5%)	10.432	0.015
Event	0 (0%)	8 (14.5%)	4 (15.4%)	7 (13.5%)		
Deaths	0	3	2	3		
Death of BC	0	2	2	2		
Death of NBC	0	1	0	0		
Lost to followup	0	5	2	4		
Median survival time	66	60	60	66	8.749	0.033
Disease-free survival	66 (100%)	46 (83.6%)	21 (80.8%)	44 (84.6%)	12.477	0.006
Event	0 (0%)	9 (16.4%)	5 (19.2%)	8 (15.4%)		
Tumor progression	0	3	3	4		
Local recurrence	0	1	0	1		
Contralateral BC	0	0	1	0		
Bone	0	0	2	0		
Hepatic	0	1	0	0		
Multiorgan	0	1	0	3		
Death of NBC	0	1	0	0		
Lost to followup	0	5	2	4		
Disease-free survival	66	60	60	66	10.850	0.013
Follow-up time					2.206	0.089
Median	33.5	30.0	39.0	33.0		
Range	25*∼*66	17*∼*65	13*∼*65	18*∼*66		

**Table 4:** Cox proportional hazards model of biological factors/treatment factors.

Parameters	Death		Tumor progression	
	Sig.	EXP(*B*)	Sig.	EXP(*B*)
Biological factors				
Age	0.024	1.089	0.034	1.076
Family history	0.924	1.054	0.850	0.905
Menopause	0.045	5.275	0.047	4.453
Histological grade	0.767	0.875	0.407	1.437
Tumor diameter	0.104	1.295	0.052	1.348
Lymph node metastasis	0.005	10.063	0.006	6.973
Cancer thrombosis	0.519	0.668	0.395	0.617
ER	0.488	0.637	0.606	0.735
PR	0.960	1.030	0.874	1.091
HER2	0.012	4.318	0.009	3.882
Ki67	0.979	0.987	0.635	1.255
P53	0.399	1.629	0.280	1.752
Treatment factors				
Operation	0.338	0.366	0.236	0.291
Chemotherapy	0.048	1.633	0.042	1.596
Radiotherapy	0.144	2.195	0.231	1.800
Endocrine therapy	0.452	0.748	0.226	0.645
